# Comparison study on antioxidant, DNA damage protective and antibacterial activities of eugenol and isoeugenol against several foodborne pathogens

**DOI:** 10.1080/16546628.2017.1353356

**Published:** 2017-07-18

**Authors:** Liang-Liang Zhang, Li-Fang Zhang, Jian-Guo Xu, Qing-Ping Hu

**Affiliations:** ^a^ School of Chemistry and Material Science, Shanxi Normal University, Linfen, China; ^b^ School of Food Science, Shanxi Normal University, Linfen, China; ^c^ School of Life Science, Shanxi Normal University, Linfen, China

**Keywords:** Eugenol, isoeugenol, antioxidant, DNA damage, antibacterial activity, flow cytometry analysis

## Abstract

Eugenol and its isomer isoeugenol are both used as flavouring agents or food additives in food products, and have both some similar biological properties. However, the difference in biological activities between eugenol and isoeugenol is rarely studied. In this study, the profiles of antioxidant, DNA-protective effects and antibacterial activities of eugenol and isoeugenol against several common foodborne pathogens were investigated and compared under various experiment conditions. Results showed that eugenol and isoeugenol had strong antioxidant activity, the protective effect against DNA damage and antibacterial activity. In addition, it was found that isoeugenol exhibited the higher biological activities mentioned above than eugenol, which was because isoeugenol had a carbon–carbon double bond closer to the benzene ring compared with eugenol. However, the specific reason needs to be further studied.

## Introduction

Environmental factors can easily lead to food oxidation while microorganisms can easily result in food poisoning and food spoilage, which is one of the most important issues facing the food industry and consumers [1]. Accompanied by growing consumer interest in natural food additives, the search for effective antioxidants and antibacterial agents from natural resources as an alternative to suppress food deterioration during food processing, transportation and storage has been reinforced, due to their presenting fewer side effects than synthetic chemicals used in today’s foods [2,3].

Eugenol (EUG, 4-allyl-1-hydroxy-2-methoxybenzene), a natural phenolic compound found in essential oils from plants including clove, cinnamon, basil, and nutmeg, has been considered non-mutagenic, non-carcinogenic and generally recognized as safe (GRAS) by Food and Drug Administration [4]. The compound exhibited a wide variety of biological properties including antioxidant [5], antibacterial [6], anti-inflammatory [7], antitermitic and antifungal activities [8]. Like eugenol, its isomer isoeugenol (ISOE, 2-methoxy-4-propenyl-phenol) is found in several spices and is used as a flavouring and storage agent, also exhibiting some similar biological properties [7,9–11]. Despite the previously described studies of eugenol and isoeugenol on diverse bioactivities, little information concerned the differences in their biological activities, even fewer studies were reported about antibacterial activity of isoeugenol against some common foodborne pathogens.

Therefore, the aim of the present study was to investigate and compare the profiles of antioxidant, DNA-protective effects and antibacterial activities of eugenol and isoeugenol against selected foodborne pathogens through a variety of antioxidant and antibacterial methods under various experiment conditions, which is very important for the preparation of eugenol isomers and their application in food and medicine.

## Materials and methods

### Chemicals

2,2-Diphenyl-1-picrylhydrazyl (DPPH), 2,6-ditert-butyl-4-hydroxytoluene (BHT), 2,2ʹ-azobis (2-methylpropionamidine) dihydrochloride (AAPH) and 2,2ʹ-azino-bis (3-ethylbenothiazoline-6-sulfonic acid) diammonium salts (ABTS) were from Sigma (USA). 2, 4, 6-Tri (2-pyridyl)-s-triazine (TPTZ), eugenol and isoeugenol were purchased from Fluka (Switzerland). Propidium iodide (PI) was from BD Biosciences. The pBR322 plasmid DNA was from Takara Bio Co. Ltd. (Dalian, China). Nutrient agar (NA) and nutrient broth (NB) mediums were from Beijing Aoboxing Bio-tech Co. Ltd. (Beijing, China). Other chemicals used were all of analytical grade.

### Microorganisms and culture

Six kinds of common food spoilage bacteria are selected in the study. Three Gram-positive strains were *Staphylococcus aureus* ATCC 25923, *Bacillus subtilis* ATCC 6051 and *Listeria monocytogenes* ATCC 19115. Three Gram-negative bacteria were *Escherichia coli* ATCC 25922, *Salmonella typhimurium* ATCC 19430, and *Shigella dysenteriae* CMCC (B) 51252. Strains were provided by the College of Life Science, Shanxi Normal University, and cultured at 37°C on nutrient agar (NA) and nutrient broth (NB) mediums.

### DPPH assay

The scavenging rate and scavenging activity of the sample on DPPH radicals were determined as described in a previous report [12]. The scavenging activity was expressed by EC_50_ value, that is the effective concentration at which free radicals are scavenged by 50% and is obtained by interpolation from regression analysis.

### ABTS assay

The ABTS cation radical scavenging activity was determined according to the method described by Xu et al. [12]. The scavenging rate and EC_50_ value were calculated using the equation described for DPPH assay.

### Ferric reducing antioxidant power (FRAP) assay

The reducing ability was determined by using FRAP assay described by Xu et al. [12]. The standard curve was constructed using FeSO_4_ solution (100–1000 μM), and FRAP value was expressed as millimoles Fe(II) per gram sample.

### Inhibition of lipid peroxidation

Lipid peroxidant value (POV) of samples was evaluated according to the method of Li et al. with some modiﬁcations [13]. The samples and BHT was dissolved in ethanol and mixed with fresh lard and put in a dark oven at 60°C respectively. A blank titration was performed parallel to treatment and POV was calculated using the following formula: peroxide value (meq of oxygen/kg) = 1000*S* × *N*/*W*. In this formula, *S* was the volume of sodium thiosulphate solution (blank corrected) in mL, *N* was the normality of sodium thiosulphate solution (0.02 N) and *W* was the weight of oil sample (gram).

### Protection of DNA oxidative damage induced by Fe^2+^

The ability of samples to protect supercoiled pBR322 plasmid DNA against Fe^2+^ and H_2_O_2_ was estimated with the DNA nicking assay as described in the previous report [12].

### Protection of DNA oxidative damage induced AAPH

The ability of samples to protect supercoiled pBR322 plasmid against AAPH was measured according to the method described by Zhang and Omaye [14] with some modifications. Intact pBR322 plasmid (0.5 µg) was mixed with various concentrations of samples and 2 µL of 25 mM AAPH in PBS (pH 7.4), and the mixture was incubated for 30 min at 37°C. Then the samples were electrophoresed on 0.8% agarose gel containing 0.5 μg/mL ethidium bromide, and photos of DNA bands were taken under gel image analysis system.

### Antibacterial activities

The sample was first dissolved and then sterilized by filtration through 0.22 μm Millipore filters. Antibacterial tests were then carried out by the Oxford cup method using 100 μL of suspension containing 10^7^ CFU/mL of bacteria spread on nutrient agar (NA) medium. Oxford cups (6 mm in diameter) were placed on the inoculated agar, and then 100 μL of sample was added with a micropipette. The diameter of zone of inhibition (ZOI) was measured after 24 h of incubation at 37°C. Tests were performed in triplicate.

### Minimum inhibitory concentration (MIC) and minimum bactericide concentration (MBC) assay

Two fold serial dilutions of samples were prepared in sterile NB medium. To each tube 100 μL of the exponentially growing bacterial cells was added to give a cell concentration of approximately 1 × 10^7^ CFU/mL. The tubes were incubated at 37°C for 24 h and then examined for evidence of the growth. The MIC and MBC were determined according to the method described by Diao et al. [15].

### Growth curve analysis

One hundred microliters of samples filtrating through 0.22 μm Millipore filters was added to 4.8 mL of the sterile NB medium, and then mixed with 100 μL of a 10 h culture of tested bacteria (1 × 10^7^ CFU/mL). The cultures were incu bated at 37°C and shaken at 120 rpm. At selected time intervals, samples from test culture were taken and the absorbance at 600 nm (OD_600_) was measured.

### Flow cytometry analysis

Logarithmic phase bacteria were collected by centrifugation at 6000×g for 5 min, washed three times, and resuspended in PBS (pH 7.4). Tested bacteria were treated with different concentrations of samples. After 0.5 and 1 h, cells containing approximately 1 × 10^8^ CFU/mL were harvested by centrifugation at 6000×g for 5 min and stained for 10 min with the equal volume of 1 mg/mL PI in the dark at room temperature. The flow cytometer (FACScan, BD Biosciences) equipped with a CellQuest software (BD Biosciences) were used to analyse 1 × 10^4^ cells after 30 min of reaction in a dark environment at room temperature. Cells were sorted into living and necrotic cells, and this assay was repeated five times.

### Statistical analysis

One-way analysis of variance (ANOVA) and Duncan’s multiple range tests were carried out to determine significant differences (*p *< 0.05) between the means by Data Processing System (DPS, version 7.05) and Excel program.

## Results

### DPPH and ABTS radicals scavenging activity

The scavenging activity of eugenol and isoeugenol on DPPH and ABTS radicals is shown in Table 1. The EC_50_ values of eugenol and isoeugenol on DPPH radicals were 22.6 and 17.1 μg/mL (*p *> 0.05), indicating that the scavenging activity of isoeugenol was slightly higher than that of eugenol. However, the scavenging activity of eugenol and isoeugenol on DPPH radicals was lower than that of Trolox (EC_50_ was 13.5 μg/mL). The profile of scavenging activity of eugenol isomers on ABTS was similar to the result of the scavenging DPPH radicals. Somewhat differently, the EC_50_ values on scavenging ABTS cation radicals were 146.5 and 87.9 μg/mL for eugenol and isoeugenol, and the scavenging activity of isoeugenol was significantly higher than that of eugenol (*p *< 0.05), which was lower than of Trolox (EC_50_ was 84.34 μg/mL). These differences in data between DPPH and ABTS assays were likely due to different experimental conditions. The disappearance of DPPH and ABTS cation radicals is directly proportional to the amount of antioxidant present in the reaction mixture. Similarly, eugenol and isoeugenol showed a concentration-dependent scavenging of the DPPH and ABTS cation radicals at certain concentrations. Their antioxidant activity in the above assays may be mediated through direct trapping of the free radicals through transfers of hydrogen or electron [16].

**Table 1.** DPPH and ABTS radicals scavenging capacity and FRAP of eugenol and isoeugenol.

	DPPH		ABTS		
	Regression equation(10–50 μg/mL)	EC_50_(μg/mL)	Regression equation(50–250 μg/mL)	EC_50_(μg/mL)	FRAP(mmol Fe(II)/g)
EUG	y = 0.245Ln(x) − 0.258	22.1 ± 3.	y = 0.134Ln(x) − 0.169	146.5 ± 5.6a	11.2 ± 1.5b
	R^2^ = 0.9995	5a	R^2^ = 0.9986		
ISOE	y = 0.286Ln(x) − 0.320	17.6 ± 4.1a	y = 0.357Ln(x) − 1.099	87.9 ± 4.7b	18.4 ± 1.2a
	R^2^ = 0.9972		R^2^ = 0.9992		

Notes: Values represent means of three independent replicates ± SD. R^2^ refer to the regression coefficients. Different letters within a column indicate statistically significant differences between the means (*p* < 0.05) for eugenol and isoeugenol.

### Ferric reducing antioxidant power (FRAP)

The FRAP may serve as a significant indicator of the potential of antioxidant activity [17]. Table 1 showed that the reducing power of eugenol and isoeugenol was 11.2 and 18.4 mmol Fe(II)/g, indicating that isoeugenol had better reducing power than eugenol (*p* < 0.05). These results suggested that eugenol isomers could result in reducing Fe^3+^/ferricyanide complex to the ferrous form (Fe^2+^), and had a remarkable potency to donate electron to reactive free radicals, transforming them into more stable non-reactive species and terminating the free radical chain reaction.

### Inhibition effects on lipid peroxidation

**Figure 1.**
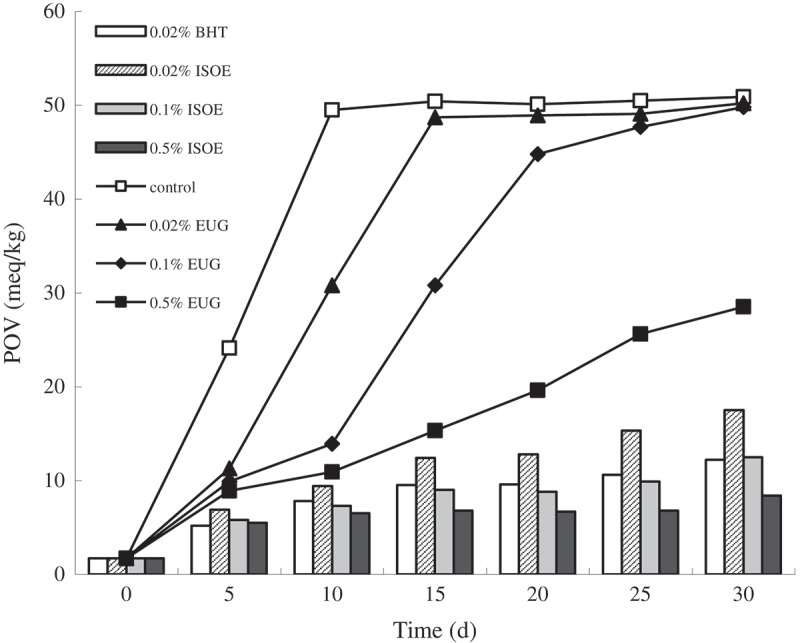
Inhibition effects of eugenol and isoeugenol on lipid peroxidation. EUG, ISOE and BHT refer to eugenol, isoeugenol and 2,6-ditert-butyl-4-hydroxytoluene respectively.

In order to investigate the inhibition effects of eugenol and isoeugenol on lipid peroxidation, the lard auto-oxidation system was tested under controlled conditions. The results showed that the POV of control increased rapidly and significantly from 1.7 to 49.5 meq/kg oil by autooxidation for 10 days, and then the POV had no significant increase (*p* > 0.05) with the prolong of treatment time and only a 2.8% increase from days 10 to 30 (Figure 1). Similar to this, the POV of 0.02% eugenol significantly increased to 48.7 meq/kg oil for 15 days, while the POV of 0.1% and 0.5% eugenol increased to 49.8 and 28.5 meq/kg oil for 30 days respectively (Figure 1). The POV of 0.02% BHT and 0.02%, 0.1%, 0.5% isoeugenol against auto-oxidation of the lipid for 30 days increased to 12.5, 17.5, 12.0 and 8.4 meq/kg oil respectively (Figure 1). All samples maintained a lower POV at 60°C than the control, and the inhibition effect was dose-dependent, increasing with higher dosage in a certain concentration range (Figure 1). At the same conditions, the POV of isoeugenol was far lower than that of eugenol, which indicated that the inhibition effect of isoeugenol against lipid peroxidation was stronger than eugenol, but weaker than BHT. The results showed that eugenol and isoeugenol can act as most potent inhibitor of lipid peroxidation, which may be attributed to its donation of a phenolic hydrogen atom to trap chain-forming peroxy radicals that induced lipid peroxidation in lard auto-oxidation system [18].

### DNA damage protective effect

**Figure 2.**
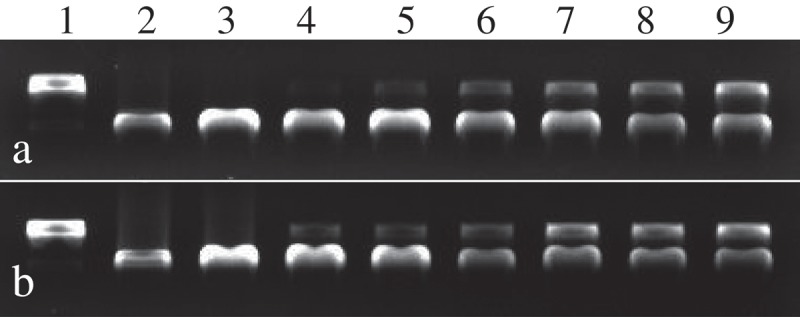
The protection effects of eugenol (a) and isoeugenol (b) on DNA oxidative damage induced by Fe^2+^ and H_2_O_2_. Lane 1, the native DNA; lane 2, the DNA treated with Fe^2+^; lane 3, the DNA treated with Fe^2+^ and solvent; lanes 4–9, the DNA treated with eugenol and isoeugenol (10, 20, 30, 40, 50, 60 mg/mL respectively).

The protection effects of eugenol and isoeugenol on DNA oxidative damage induced by Fe^2+^ and H_2_O_2_ were evaluated and the results are shown in Figure 2. From the gel analysis, eugenol and isoeugenol showed effective and concentration dependent reduction in the formation of nicked DNA and increased super coiled form of DNA. In concentration range from 10 to 60 mg/mL, the DNA damage protective effects of different concentrations of eugenol were 2.8%, 5.6%, 19.1%, 26.4%, 42.2% and 48.8% respectively, while the protection effects of isoeugenol were 7.5%, 11.5%, 29.6%, 45.3%, 48.2% and 58.6% respectively. The results indicated that the isoeugenol possessed higher DNA damage protective effect than eugenol, and that eugenol and isoeugenol might prevent the Fenton’s reaction, and or it probably quenched hydroxyl radicals by donating hydrogen-atom or electron [19].

**Figure 3.**
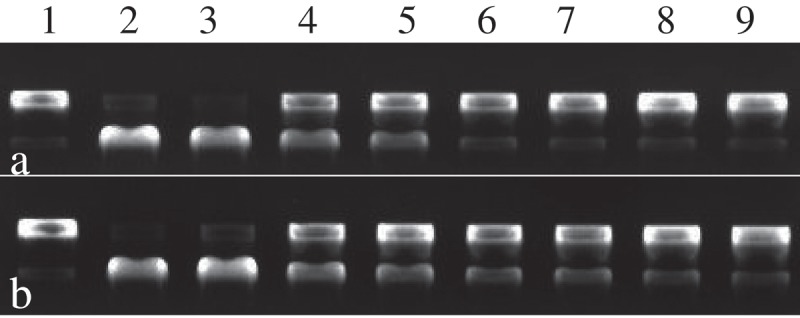
The protection effects of eugenol (a) and isoeugenol (b) on DNA oxidative damage induced by AAPH. Lane 1, the native DNA; lane 2, the DNA treated with AAPH; lane 3, the DNA treated with AAPH and solvent; lanes 4–9, the DNA treated with eugenol and isoeugenol (20, 40, 60, 80, 100, 120 μg/mL respectively).

In order to further evaluate the abilities to protect DNA damage of eugenol and isoeugenol, the protective effect assay of DNA from oxidative damage of AAPH was carried out. Supercoiled plasmid DNA (Figure 3, lane 1) was prone to oxidation by peroxyl radicals generated by AAPH, which resulted in the formation of open circular (Figure 3, lane 2). As shown in Figure 3, similar results were found in protective effect assay of DNA from oxidative damage of AAPH. In concentration from 20 to 120 g/mL, the DNA damage protective effects of different concentrations of eugenol were 46.7%, 60.8%, 68.3%, 72.1%, 89.0% and 91.7% respectively, while the protection effects of isoeugenol were 58.1%, 73.5%, 91.2%, 94.4%, 95.6% and 96.6%. The protection offered against DNA damage of AAPH by eugenol and isoeugenol was dose-dependent, increasing with higher dosage. The differences in protective effect between two DNA-damage assays for eugenol or isoeugenol may come from different determination method [20]. Nonetheless, these findings showed that the eugenol and isoeugenol owned a higher potential to prevent DNA damage.

### ZOI, MIC and MBC of eugenol and isoeugenol

The ZOI, MIC and MBC values of eugenol and isoeugenol are presented in Table 2. The results showed that eugenol and isoeugenol had strong antibacterial activity on all of the tested bacteria. The ZOI values of eugenol and isoeugenol were in the range of 12.7–22.3 mm and 18.0–26.0 mm for all tested bacterial strains respectively. The ZOI values of isoeugenol were greater than that of eugenol, and there was a significant difference for Gram-positive bacteria; however, no difference in ZOI values for Gram-negative bacteria was found between them. The MIC and MBC values of eugenol were the equal, 312.5 µg/mL for *E. coli* and *S. dysenteriae and* 625 µg/mL for others, while the MIC and MBC values of isoeugenol were 312.5 µg/mL for each tested bacterium. These results indicated isoeugenol possessed stronger antibacterial activity than eugenol for Gram-positive bacteria and *S. typhimurium*. In order to further investigate antibacterial properties of eugenol and isoeugenol, a Gram-positive strain *L. monocytogenes* and a Gram-negative strain *E. coli* were selected as the model organisms for subsequent study.

**Table 2.** ZOI, MIC, and MBC of eugenol and isoeugenol.

	ZOI (mm)^a^		MIC (µg/mL)		MBC (µg/mL)	
Microorganisms	Eugenol	Isoeugenol	Eugenol	Isoeugenol	Eugenol	Isoeugenol
Gram-positive						
*S. aureus*	12.7 ± 0.6 cB	20.8 ± 0.6 bA	625.0	312.5	625.0	312.5
*B. subtilis*	15.3 ± 0.7 bcB	21.3 ± 2.1 bA	625.0	312.5	625.0	312.5
*L. monocytogenes*	22.3 ± 1.4 aB	26.0 ± 1.5 aA	625.0	312.5	625.0	312.5
Gram-negative						
*E. coli*	17.1 ± 1.2 bA	18.0 ± 1.8 bA	312.5	312.5	312.5	312.5
*S. typhimurium*	20.1 ± 1.0 aA	21.2 ± 1.2 bA	625.0	312.5	625.0	312.5
*S. dysenteriae*	17.0 ± 1.0 bA	18.3 ± 1.2 bA	312.5	312.5	312.5	312.5

Notes: aValues represent means of three independent replicates ± SD. Mean values within a column with different lower case letters are significantly different at *p* < 0.05 for different tested microorganisms. Mean values within a row with different upper case letters are significantly different at *p* < 0.05 for eugenol and isoeugenol.

### Growth curve analysis

**Figure 4.**
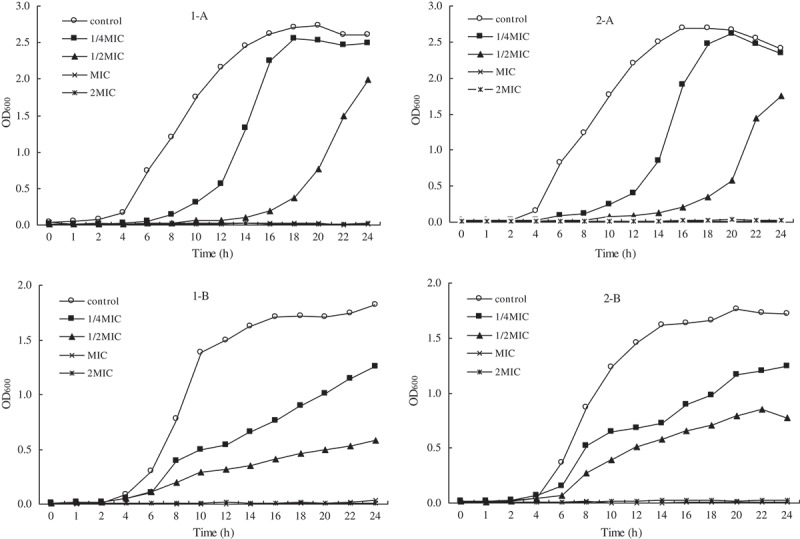
Effect of eugenol (1) and isoeugenol (2) on the viability of *L. monocytogenes* (A) and *E. coli* (B).

As observed in Figure 4, different concentrations of eugenol and isoeugenol had significant effects on growth of tested bacteria. The untreated *E. coli* started to propagate rapidly after cultured for 2 h. Compared to the control, *E. coli* treated with eugenol and isoeugenol at the 0.25× and 0.5 × MIC value showed a significant increase in the absorbance value until cultured for 6 h and 12 h (Figure 4). By contrast, the absorbance values of treatment at 1× and 2 × MIC had no change during 24 h of incubation, indicating that the growth of *E. coli* was inhibited completely. Similar results were also found for *L. monocytogenes*. These findings confirmed the inhibiting capacity of eugenol and isoeugenol on the growth rate of surviving *E. coli* and *L. monocytogenes*, and also suggested that antibacterial effects were influenced by incubation time and concentration.

### Flow cytometry analysis

**Figure 5.**
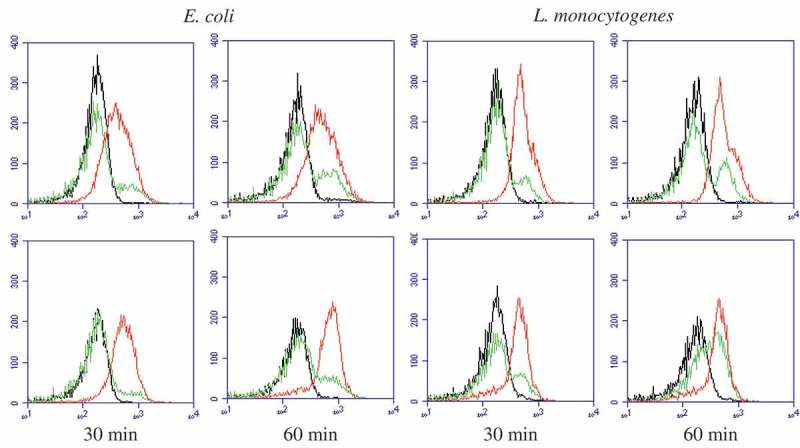
Flow cytometry images of *E. coli* and *L. monocytogenes* respectively treated with eugenol (upper panel) and isoeugenol (lower panel) for 30 min and 60 min. Black line, control; green line, 1 × MIC; red line: 2 × MIC.

PI, an intercalating agent generally excluded from viable cells, is usually used to confirm cell viability [21]. Figure 5 showed the cell viability results for various concentrations of eugenol and isoeugenol cultured with *E. coli* and *L. monocytogenes* for 30 min and 60 min respectively. The necrosis rate of tested bacterial cells changed significantly with concentrations and the time. Compared to control, the necrosis rate of *E. coli* treated with 1× and 2 × MIC eugenol significantly increased from 0.7% to 10.9% and 53.6% for 30 min (p < 0.05), and increased from 1.2% to 23.4% and 67.2% for 60 min (p < 0.05) respectively. And under the same conditions, the necrosis rate of *E. coli* treated with isoeugenol dramatically increased to 14.7% and 80.8% for 30 min (p < 0.05) and increased to 28.6% and 92.7% for 60 min (p < 0.05) respectively. As expected, similar results were observed for *L. monocytogenes* cells treated with eugenol and isoeugenol after incubation with different times (Figure 5), and what is different was necrosis rate at same concentration and time point, which probably come from the difference in genetic and growth characteristics of different strains. In general, these results suggested that eugenol and isoeugenol could dose-dependently and time-dependently induce tested bacterial death, and that isoeugenol was more effective than eugenol in antibacterial activity against *E. coli* and *L. monocytogenes*, which confirmed the above research results.

## Discussion

Eugenol and isoeugenol are the members of the phenylpropanoids class of chemical compounds, and there have been some studies on their antioxidant activity respectively [4,5,9,10]. In the present study, in order to investigate and compare comprehensively antioxidant activities of eugenol and isoeugenol, the scavenging activities of DPPH and ABTS radical, reducing power and lipid peroxidation involved in oxidative stress were respectively performed because the use of any single method for measurement of antioxidant activity can yield rather misleading results [16]. Bortolomeazzi et al. reported that eugenol possessed higher DPPH radical scavenging ability than isoeugenol [10], while Ito et al. suggested that only a little difference in the DPPH radical-scavenging rate was observed between eugenol and isoeugenol [22]. Even Nam et al. demonstrated the DPPH radical-scavenging activity of eugenol decreased with the increase of concentration [5], which was disagreement with our results. In fact, oxidation of eugenol is thought to be affected by light, enzymes and molecular oxygen, as well as by the nature of this compound [9], and thus these results cannot be compared because of differences in experimental conditions and expression ways of result. Few studies have investigated the ABTS radical-scavenging activity of eugenol and isoeugenol. The present study revealed the eugenol and isoeugenol possessed good ABTS radical-scavenging activity, and further confirmed that the antioxidant activity may be attributable to its hydrogen atom and electron-donating ability. Nam et al. reported that the reducing power was increased in dose dependent manner in the presence of eugenol [5], which was confirmed by the present results. The reducing power of eugenol and isoeugenol indicated that they possessed antioxidative potential by breaking the free radical chain, donating a hydrogen atom. Free radical scavenging is a generally accepted mechanism for antioxidants to inhibit lipid peroxidation. Ito et al. suggested that iron-mediated lipid peroxidation and autooxidation of Fe^2+^ ion were inhibited markedly by isoeugenol, and less effectively by eugenol, while copper-dependent oxidation of low-density lipoprotein (LDL) was potently inhibited by eugenol and isoeugenol to the same extent [22]. The present results showed that eugenol and isoeugenol exhibited strong inhibitory effects against lipid peroxidation induced by peroxyl radical in lard auto-oxidation system, which may be attributable to its hydrogen-donating ability.

Reactive oxygen species (ROS) are a major source of oxidative stress in cells and are generated by external and internal factors, resulting in the damage of proteins, carbohydrates, lipids and DNA. More and more evidence suggested that oxidative breakage of mammalian cellular DNA led to cell death, tissue damage and a wide array of neurological and pathological disorders [23]. Previously, Yogalakshmi et al. revealed that eugenol pretreatment prevented DNA strand break induced by thioacetamide [4]; the protective effect of eugenol against DNA oxidative damage induced by Fe^2+^ and H_2_O_2_ has been reported by Nam et al. [5], which was supported by our results. Besides, the present study found that isoeugenol can more effectively protect oxidative damage of DNA than eugenol. In addition, the abilities to protect DNA from the damage by AAPH of eugenol and isoeugenol were investigated firstly in the present study, and similar results are obtained as well, indicating that they had the scavenging activity on peroxyl radicals generated by AAPH.

From the above results, eugenol and isoeugenol exhibited better antioxidant activities and DNA damage protective effect, which may be mediated through direct trapping of the free radicals or inhibiting the propagation of radical chain reactions through transfers of hydrogen or electron [18]. The transfers of hydrogen or electron from antioxidant to free radicals and other oxidants occurred at different redox potentials and also depended on the structure of the antioxidant [24,25]. The antioxidant activities of eugenol and isoeugenol differed strongly from each other, and isoeugenol exhibited remarkably higher antioxidant activities and DNA damage protective effect than eugenol, which may come from subtle differences in their structure. Structurally, one of the oxygen’s lone pairs presenting in phenolic oxygen of both eugenol and isoeugenol overlapped with the aromatic system, which resulted in its delocalization spreading over the whole ring to some extent. As a result, the σ-bond electrons between the oxygen atom and the hydrogen atom shifted to the oxygen atom and made it easier to dissociate the hydrogen ion. And the ortho-methoxy group in these two compounds had strong electron-donating ability, which could provide them higher antioxidant activity. Theoretically, compared with eugenol, isoeugenol had a carbon–carbon double bond closer to the benzene ring, which would bring about a stronger biological activity. However, the specific reason needs to be further studied. In addition, some studies reported eugenol and isoeugenol with various concentrations possessed the antioxidant as well as pro-oxidant activities under certain circumstances [9,26,27]; however, no pro-oxidant activity was found under the present test conditions.

Some studies have reported the antibacterial activity of eugenol against some bacteria [6,28–30], but there was very little information on isoeugenol in this respect. In this study, the antibacterial activities of eugenol and isoeugenol against several common foodborne pathogens were investigated primarily. It was found that both eugenol and isoeugenol had strong antibacterial activity on the basis of ZOI, MIC and MBC assays, and incubation time and concentration presented significant inhibitory effects on the growth of surviving *E. coli and L. monocytogenes* based on growth curve analysis and flow cytometry assays. Expectedly, similar to results of the antioxidant activity, isoeugenol also exhibited stronger antibacterial activity than eugenol as a whole. The phenolic hydroxyl group had weak acidity, which partly resulted in its inhibitory activity on bacteria. In addition, polyphenols perform multiple biological functions and many of these functions have been attributed to its antioxidant activity [31], and a positive correlation between antioxidant and antibacterial activity was found in this study. Therefore, the difference in antibacterial activity between eugenol and isoeugenol was likely to be related to their antioxidant activity and structure as well.

## Conclusions

In conclusion, it is clear that eugenol and isoeugenol had an excellent reducing power and exerted antioxidant activity against DPPH, ABTS, lipid peroxidation, and possessed the protective effect against DNA damage induced by hydroxyl radical and AAPH, as well as antibacterial activities against several common foodborne pathogens. Their antioxidant activity and DNA damage protective effect were dose-dependent, increasing with a higher dosage in a certain concentration range, and the antibacterial effects were significantly influenced by incubation time and concentration. In addition, it was found that isoeugenol exhibited a higher antioxidant activity, DNA damage protective effect and antibacterial activity than eugenol, which was probably because isoeugenol had a carbon–carbon double bond closer to the benzene ring compared with eugenol, indicating that the biological activity of eugenol and isoeugenol mainly came from their structure, while subtle differences in the structure of eugenol isomers can lead to obvious change in their biological activity.
